# Overestimates of Survival after HAART: Implications for Global Scale-Up Efforts

**DOI:** 10.1371/journal.pone.0001725

**Published:** 2008-03-05

**Authors:** Gregory P. Bisson, Tendani Gaolathe, Robert Gross, Caitlin Rollins, Scarlett Bellamy, Mpho Mogorosi, Ava Avalos, Harvey Friedman, Diana Dickinson, Ian Frank, Ndwapi Ndwapi

**Affiliations:** 1 University of Pennsylvania School of Medicine, Philadelphia, Pennsylvania, United States of America; 2 Botswana-Harvard School of Public Health AIDS Initiative Partnership for HIV Research and Education, Gaborone, Botswana; 3 Infectious Disease Care Clinic, Princess Marina Hospital, Gaborone, Botswana; 4 Independence Surgery, Gaborone, Botswana; Duke University Medical Center, United States of America

## Abstract

**Background:**

Monitoring the effectiveness of global antiretroviral therapy scale-up efforts in resource-limited settings is a global health priority, but is complicated by high rates of losses to follow-up after treatment initiation. Determining definitive outcomes of these lost patients, and the effects of losses to follow-up on estimates of survival and risk factors for death after HAART, are key to monitoring the effectiveness of global HAART scale-up efforts.

**Methodology/Principal Findings:**

A cohort study comparing clinical outcomes and risk factors for death after HAART initiation as reported before and after tracing of patients lost to follow-up was conducted in Botswana's National Antiretroviral Therapy Program. 410 HIV-infected adults consecutively presenting for HAART were evaluated. The main outcome measures were death or loss to follow-up within the first year after HAART initiation. Of 68 patients initially categorized as lost, over half (58.8%) were confirmed dead after tracing. Patient tracing resulted in reporting of significantly lower survival rates when death was used as the outcome and losses to follow-up were censored [1-year Kaplan Meier survival estimate 0.92 (95% confidence interval, 0.88–0.94 before tracing and 0.83 (95% confidence interval, 0.79–0.86) after tracing, log rank P<0.001]. In addition, a significantly increased risk of death after HAART among men [adjusted hazard ratio 1.74 (95% confidence interval, 1.05–2.87)] would have been missed had patients not been traced [adjusted hazard ratio 1.41 (95% confidence interval, 0.65–3.05)].

**Conclusions/Significance:**

Due to high rates of death among patients lost to follow-up after HAART, survival rates may be inaccurate and important risk factors for death may be missed if patients are not actively traced. Patient tracing and uniform reporting of outcomes after HAART are needed to enable accurate monitoring of global HAART scale-up efforts.

## Introduction

As of 2005 an estimated 1.3 million people were receiving highly active antiretroviral therapy (HAART) in low- and middle-income countries[1]. As HAART scale-up has progressed, major challenges inherent in following large numbers of patients in resource-limited settings have emerged[2]. One problem relates to keeping patients in care, and published rates of loss to follow-up after HAART initiation in antiretroviral therapy programs in sub-Saharan Africa have varied widely[3], from approximately 0[4] to 39%[5]. Furthermore, while patient tracing (e.g., phoning or visiting patients when they do not present to clinic) is ideal it is not always feasible and passive follow-up, where patients who miss visits are not traced, is common.

From a public health perspective, the most important measure of an antiretroviral therapy program's effectiveness is survival after HAART. However, losses to follow-up may threaten the validity of analyses of survival if the proportion of patients dying after HAART is high and only known deaths are counted as events. Furthermore, censoring, or not counting, patients lost to follow-up as having died, while standard, could lead not only to inaccurate estimates of survival but to biased estimates of risk factors for death as well. The latter issue, called informative censoring, may occur in survival analyses when patients who are lost to follow-up are both censored and at high risk of having died[6]. Since many cohort studies of survival after HAART have had substantial rates of losses to follow-up after HAART[3], and since both monitoring antiretroviral therapy scale-up efforts and improving these efforts depends on accurate reporting of survival rates and risk factors for death, evaluating and quantifying these issues is of global health importance. To investigate this further, we analyzed how outcomes and risk factors for death after HAART initiation in a large public treatment program in sub-Saharan Africa would be reported in two scenarios: one before and one after patient tracing.

## Methods

### Study Design

This was a cohort study comparing treatment outcomes and risk factors for death within the first 12 months after HAART initiation as reported before and after patient tracing during early rapid scale-up within Botswana's National Antiretroviral Therapy Program (called Masa, which is Setswana for “New Dawn”). Since determining outcomes on patients within a clinic is conceivably most difficult during initial rapid scale-up, and since Botswana's National Antiretroviral Therapy Program officially began in 2002, we studied patients presenting for HAART soon after the clinic began dispensing HAART.

### Study Setting

This study was set at one of the main public clinics of Masa, the Infectious Disease Care Clinic (IDCC), in Gaborone, Botswana. The IDCC in Gaborone was the first clinic of Masa to begin providing HAART to patients with AIDS and, during the study period, was the main site of rapid HAART scale-up efforts in the country. Thus, early in Botswana's scale-up effort, the IDCC encountered challenges with respect to providing HIV care to an increasingly large number of individuals with limited resources, and therefore was considered an appropriate site in which to perform the study.

Patients presenting to Masa for care routinely have data on a patient or patient contact's phone number and information regarding place of current residence collected at the time of registration. In Masa, patients required either a CD4 count of less than 200 cells/mm^3^ or an opportunistic infection to start HAART. In Masa at the time the data were collected, CD4 counts were the primary variable used to time initiation of HAART and thus World Health Organization and Center for Disease Control and Prevention (CDC) staging were usually unavailable for analysis. Tuberculosis in Botswana is considered present if the patient has a history of a positive acid fast bacilli smear from any site or has a negative sputum smear and a positive culture for *M. tuberculosis* or highly suspicious radiological features or clinical findings of tuberculosis as determined by a physician [7]. First line therapy in Masa during the study consisted of fixed dose combination zidovudine and lamivudine (Combivir®) plus efavirenz (Sustiva®) or nevirapine (Viramune®). Patients were seen at a pretreatment visit and adherence counseling and standard baseline tests were done including CD4 count (EPICS, Beckman Coulter), plasma HIV-1 RNA level (Amplicor HIV-1 Monitor Assay), and complete blood count. Patients then initiated HAART and were followed via clinical visits approximately every 3 months and medications were filled on a monthly basis, most often coinciding with a clinic visit, when possible.

### Study Subjects

We retrospectively identified all HIV-infected adults (≥18 years) consecutively presenting to the IDCC for care and initiating HAART during the study period of interest using the Microsoft Access^©^ database which was designed for use in clinical care at the IDCC during this time. Due to the implementation of a new national electronic medical record in March 2004, which resulted in cessation of use of the Access database, and because we wanted each patient to have at least 6 months of potential follow-up, patients were eligible if they registered at least 7 months prior to March 2004 (the extra month was included to give patients time to register and, two to four weeks later, initiate HAART). Thus, patients registering at the IDCC between February 2003 and August 2003 and initiating HAART were eligible for inclusion in the study.

### Data Collection

After identification of eligible study subjects, we used the IDCC paper chart, on which primary caregiver notes are written, and the IDCC electronic database in use at the time, which also contains clinical notes as well as information on dates of patient clinic and hospital visits and pharmacy data, to retrospectively describe patient characteristics. Providers record clinical data in these sources during patient visits. Tuberculosis was considered present if the paper or electronic records noted this condition or if the patient was on anti-tubercular therapy at the time of HAART initiation (as indicated by either the clinical or pharmacy record). Information from these sources was later abstracted and entered into a study-specific Microsoft Access database.

Each patient in the cohort was initially classified as alive, lost, or dead using this retrospective data collection process. Specifically, a patient was considered alive and in care if they were in between visits and were known to have refilled their HAART at the pharmacy within the prior 30 days, and as dead if their deaths were recorded anywhere in the clinical record (including records of the adjacent tertiary care hospital). Before tracing, a patient was classified as lost if their last contact with the clinic (including the pharmacy) was greater than 30 days past their last scheduled visit.

Patients who were lost to follow-up at this stage were prospectively traced. Patient tracing involved phone calls and, if necessary, home visits. Maps to patients' houses were not routinely collected at the time of registration. Specifically, patients initially classified as lost to follow-up were searched for by first calling the available contact number provided to the clinic at the time of registration and, if that did not result in useful contact, calling patient contacts, also provided at registration. If after 3 phone calls on variable times on separate days the patient's status could not be ascertained, a Setswana-speaking study nurse from the IDCC made a home visit to check with either the patient or his family. Patient transfers to other clinics were recorded in the clinic database and were ascertained if present. If the phone calls and home visits neither confirmed that the patient was alive nor confirmed their death, the patient remained classified as lost to follow-up. Dates of death were obtained from the paper or electronic clinical record or, if not in that source, from patient contacts. Patients who were alive and in care and those who were lost to follow-up were classified as having their respective outcomes at the date of the last clinic visit. Due to the retrospective nature of patient identification, patient tracing was performed at variable time intervals after a patient's last clinic visit.

### Statistical Analysis

Continuous variables were compared using t tests or rank sum tests after testing the normality of the distribution using the Shapiro-Wilk test. Chi-square tests were used to compare categorical variables.

Date of HAART initiation and date of outcome were used as the start and end point of follow-up time, respectively. Patients who were alive and in care and patients who were lost to follow-up were censored as of the date of their last clinic visit. Kaplan-Meier plots were used to present 1-year survival estimates before and after patient tracing. Risk factors for death before and after patient tracing were evaluated using Cox proportional hazards models after testing the validity of the proportional hazards assumption[6], [8]. Factors where the 95% confidence interval of the point estimate for the unadjusted relative hazard did not cross 1 on unadjusted analysis were retained and evaluated in a multivariable model. However, in order to evaluate if the inclusion of possible confounders not meeting this criterion in the analysis affected the study's findings, we also evaluated the results after forcing variables plausibly associated with survival in the multivariable model [9]. Collinearity between potential risk factors was assessed by examining the standard errors for the hazard ratios when the multivariable Cox regression model was fitted. Bias was considered present if the point-estimates of the relative hazards produced before and after tracing differed by 20% or more[6]. Analyses were conducted using STATA version 8.2 (Intercooled).

The Health Research Development Council of the Botswana Ministry of Health and the Institutional Review Board of the University of Pennsylvania approved this study. Since tracing in the case of a patient becoming lost to follow-up is discussed with patients initiating HAART in the IDCC, a waiver of informed consent was obtained for the study.

## Results

### Patient characteristics

Between February 5^th^ and July 29^th^, 2003, a total of 524 patients presented to the IDCC for evaluation to start HAART. Of these, 410 (78%) initiated HAART during the study period and therefore were included in subsequent analyses. Of the 114 who were not included in the study, 38 (33%) were transferred to other clinics prior to HAART initiation, 32 (28%) had CD4 counts >200 cells/mm^3^ and no opportunistic infections and therefore did not qualify for HAART, 17 (15%) died shortly after registration and prior to initiating HAART, 12 patients (11%) initiated HAART but would not have had at least 6 months of follow-up and were therefore excluded, 12 (11%) were lost to follow-up shortly after registration and prior to initiating HAART, and 3 (3%) patients had tuberculosis and CD4 counts >500 cells/mm^3^ and therefore had HAART delayed.

The baseline characteristics of the 410 patients initiating HAART are given in Table 1. Most patients [339 of 410, (83%)] initiated regimens containing fixed-dose zidovudine and lamivudine plus a non-nucleoside reverse transcriptase inhibitor. 359 of 410 (88%) were treatment naïve at the time of initiation.

**Table 1:** Baseline characteristics of patients prior to initiating HAART in the IDCC, Gaborone, Botswana (N = 410)

Age: years, mean (range)	37 (19–74)
Female sex, n (%)	244 (60%)
Weight: kg, median (IQR)	52 (45–60)
ARV naïve, n (%)	359 (88%)
CD4 count, median cells/mm^3^ (IQR)	81 (31–145)
Viral load, median log_10_ copies/mL plasma (IQR)	5.67 (5.11–5.88)
Hemoglobin level, g/dL (IQR)	10.3 (8.9–11.8)
Tuberculosis, n (%)	133 (32%)
Initial HAART regimens	
zidovudine+lamivudine+efavirenz	171 (42%)
zidovudine+lamivudine+nevirapine	168 (41%)
lamivudine+stavudine+efavirenz	24 (6%)
lamivudine+stavudine+nevirapine	23 (5%)
didanosine+stavudine+efavirenz	3 (1%)
didanosince+stavudince+nevirapine	8 (2%)
Other	14 (3%)

IQR  =  inter-quartile range; ARV  =  antiretroviral therapy; HAART  =  highly active antiretroviral therapy

### Analysis of outcomes before and after patient tracing

**Figure 1:**
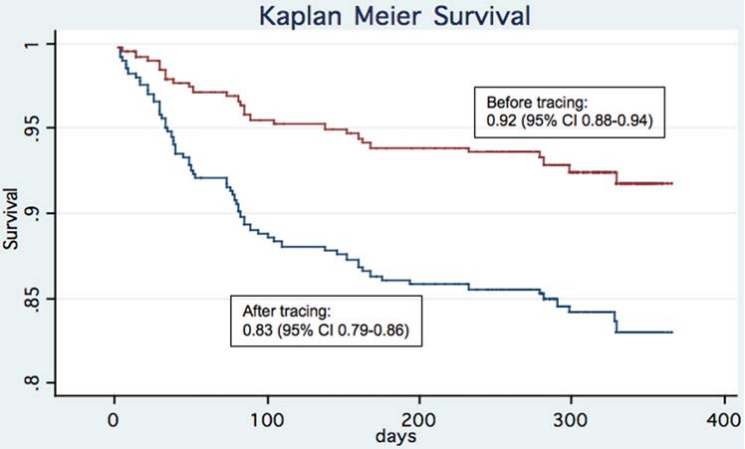
Kaplan-Meier curve 52-week survival estimates before and after patient tracing, IDCC, Gaborone, Botswana. Losses to follow-up are censored. Log rank P <0.001.

410 patients contributed 317 patient-years of follow-up; the median duration of follow-up among patients on HAART was 44 weeks (inter-quartile range (IQR), 37–49). Of the 68 patients who were originally lost to follow-up, 65 (96%) were called and 19 (28%) were visited. Patient tracing significantly increased the number of reported deaths [29 of 410 (7.1%) vs. 69 of 410 (16.8%); P<0.001 for difference in proportion reported dead]. Of the 68 patients initially categorized as lost, over half (58.8%) were confirmed dead after tracing (Table 2), and 57.9% (40 of 69) of all deaths after HAART were initially categorized as lost to follow-up. The median survival time after HAART for those who were characterized as lost before tracing and then confirmed dead after tracing was 47 days (IQR, 24–98 days). Furthermore, the median pre-treatment CD4 cell count of the 22 patients who remained lost to follow-up after tracing was significantly lower compared to pre-treatment CD4 counts of patients remaining alive and in care [37 cells/mm^3^ (IQR 17–77) vs. 98 (IQR 38–158), rank sum P = 0.009]. The 1-year Kaplan-Meier survival estimates before and after tracing are shown in Figure 1. Patient tracing resulted in reporting of a significantly lower estimate of survival than was observed before tracing [log rank P<0.001, Figure 1]. This effect can be attributed to an increased number of ascertained deaths in the setting of an unchanged number of patients at risk after tracing (Table 2).

**Table 2:** Patient outcomes before and after tracing in the Infectious Disease Care Clinic, Gaborone, Botswana.

Outcome	Before tracing (95% CI)	After tracing (95% CI)	P value*
Lost: n, %	68 (16.6%, 13.1–20.5)	22 (5.4%, 3.4–8.0)	<0.001
Dead: n, %	29 (7.1%, 4.7–10.0)	69 (16.8%, 13.3–20.8)	<0.001
Alive on HAART: n, %	313 (76.3%, 71.2–80.3)	319 (77.8%, 73.5–81.7) †	>0.5

N = 410

* P value is for difference in proportion of patients categorized as lost, dead, or on HAART according to two different methods of follow-up.

† Tracing revealed that 6 patients originally categorized as lost were still on HAART in the IDCC but had different medical record numbers and were not included in the pharmacy database.

### Risk factors for death after HAART

All variables in Table 1 were analyzed in univariate analysis. Using outcomes obtained by tracing, patients who died at any time during the period of study were significantly more likely to be male [36 of 166 (21.7%) vs. 33 of 244 (13.5%); P = 0.03] and to have lower baseline CD4 counts [median 33.5 (IQR 15–75) vs 94.0 cells/mm^3^ (IQR 38–155), P<0.001] and lower baseline hemoglobin levels [median 9.8 gm/dL (IQR 7.9–11.1) vs 10.4 gm/dL (IQR 9.1–11.9), P = 0.002]. Men had lower baseline CD4 counts than women [median 68 (IQR 29–116) vs. 90 (IQR 32–160) cells/mm^3^, P = 0.03)] but higher baseline hemoglobin levels [median 9.9 (IQR 8.6–11.1) vs. 11.1 gm/dL (IQR 9.5–12.6), P <0.001] and similar plasma viral loads [5.7 (IQR) 5.2–5.9) vs. 5.6 (IQR 5.1 vs. 5.9) log_10_ copies/mL, P  = 0.37]. Unadjusted hazard ratios for these variables are presented in Table 3. Categorization of baseline CD4 count and baseline hemoglobin levels as shown in Table 3 is based on the finding that risk did not vary substantially across finer categorization schemes (data not shown). Adjusted hazard ratios (AHR), controlling for male sex, baseline CD4 count, and baseline hemoglobin level (which were independently associated with death) as well as baseline viral load and age (which did not meet our criteria for potential confounders but which but which are plausibly associated with survival and were therefore forced into the model) are also presented in Table 3.

**Table 3:** Hazard ratios for death before and after tracing after initiation of HAART in the IDCC, Gaborone, Botswana.

	Before tracing		After tracing	
	Unadjusted HR (95% CI)	Adjusted HR (95% CI)	Unadjusted HR (95% CI)	Adjusted HR (95% CI)
**Characteristic**				
Sex				
Female	1		1	
Male	1.40 (0.67–2.95)	1.41 (0.65–3.05)*	1.81 (1.11–2.93)	1.74 (1.05–2.87) *
Baseline CD4 count, cells/mm^3^				
≥100	1		1	
50–99	2.01 (0.61–6.60)	1.81 (0.55–6.04)†	2.80 (1.30–6.06)	2.51 (1.15–5.48) †
<50	4.06 (1.60–10.29)	3.49 (1.33–9.17)	4.60 (2.41–8.77)	3.86 (1.98–7.53)
Baseline hemoglobin, gm/dL				
≥9.0	1		1	
<9.0	1.93 (1.17–3.20)	1.76 (1.05–2.96)Ψ	1.61 (0.73–3.57)	1.44 (0.63–3.29) Ψ

N = 410

* Adjusted for baseline CD4 count (categorized as above), baseline viral load (dichotomized at 100,000 copies/mL), presence of anemia (categorized as above), and age;

† adjusted for age, baseline viral load, male sex, and presence of anemia;

Ψ adjusted for baseline CD4 count, age, baseline viral load, and male sex

HR = hazard ratio; CI = confidence interval

Bias towards no association was present for all variables tested when outcomes observed prior to tracing were analyzed, and this effect was strongest when determining the AHR of death for a baseline CD4 count of 50–99 compared to ≥100 cells/mm^3^ (AHR of 1.81 before tracing compared to 2.51 after tracing, or a 39% change) and for male sex (AHR of 1.41 before tracing compared to 1.74 after tracing, or a 23% change) (Table 3).

## Discussion

This analysis from a large public antiretroviral therapy program in sub-Saharan Africa documents a substantial death rate among patients lost to follow-up soon after initiating HAART, and illustrates how deaths among patients lost to follow-up can result in both inaccurate estimates of survival and biased estimates of risk factors for death after HAART initiation.

From a clinical perspective, the fact that nearly 60% of those initially considered lost to follow-up prior to tracing were later found to have died suggests that interventions designed to decrease mortality after HAART initiation will need to include methods to identify, locate, diagnose and, if possible, treat incident illnesses in those who miss even a single clinic visit. Given the very short survival after HAART initiation among patients initially categorized as lost but who were eventually confirmed dead (i.e., 42 days), this finding also suggests that such interventions should be able to identify these patients rapidly. From a public health perspective, these results suggest that death rates after HAART initiation within antiretroviral therapy clinics may be systematically underestimated if losses to follow-up are substantial and patients with very low pre-treatment CD4 counts are presenting for care. For example, in this study, nearly 60% of all deaths within the first year would not have been detected unless patient tracing was performed. Thus, precise estimates of the actual effectiveness of global antiretroviral scale-up efforts will be difficult if not impossible to obtain unless patient tracing is undertaken, at least among those on whom outcomes are formally reported.

Since limited data on definitive outcomes among patients lost to follow-up after HAART initiation in sub-Saharan Africa exist, it is difficult to assess the scope of this problem. In particular, although patient tracing has been associated with lower survival estimates among HIV-infected patients not on HAART[10], little is known about actual outcomes among patients who have initiated HAART. However, a recent report from four public sector antiretroviral therapy clinics in northern Malawi during 2004–2006 used patient tracing (including home visits) and found that 50% of those not attending clinic for 3 or more months had died, indicating that high rates of death among patients who do not return to clinic after HAART initiation are also currently being found in other settings[11]. Since only 5% of all patients starting HAART in the Malawi report were categorized as lost to follow-up, however, the effects of deaths among those initially considered lost on overall survival estimates would likely have been less pronounced than was shown in this study, where the initial loss to follow-up rate was approximately 17%[11]. This study extends findings from the Malawi study by documenting the effect of undetected deaths on overall survival estimates, and suggests that the magnitude of this effect likely relates both to the overall loss to follow-up rate and the proportion of deaths among these individuals. Since losses to follow-up in several large public antiretroviral therapy clinics in Zambia (21%)[12], South Africa (14%), Cote d'Ivoire (11%)[13], additional settings in Malawi (7%)[14], [15], Uganda (11%)[16], Kenya (15–25%)[17], [18] and in the Antiretroviral Therapy in Lower Income Countries (ART-LINC) collaboration (19% among clinics not performing tracing)[19] have been substantial, underestimation of deaths after HAART initiation in many reports from the region could be common.

The finding of substantial death rates among patients who are lost to follow-up also suggests that death rates after HAART initiation in the developing world may be higher than previously suspected. The ART-LINC study comparing outcomes after antiretroviral therapy initiation in low and high income countries excluded clinics that did not trace patients from survival analyses, found greater ascertainment of deaths in developed countries, and documented higher rates of losses to follow-up in developing countries[19]. Thus, while inability to ascertain outcomes among patients lost to follow-up could bias mortality estimates in both the ART-LINC and the ART-CC data, higher rates of losses to follow-up in the ART-LINC patients creates the possibility that deaths may have been underestimated to a greater degree in this group.

This study differs from several previous reports in that we performed prospective data collection on patients consecutively initiating HAART at a large antiretroviral therapy program in sub-Saharan Africa specifically to determine definitive outcomes on patients who were initially considered lost to follow-up and to compare survival estimates and risk factors for death before and after active tracing was performed. Although the ART-LINC collaboration documented higher rates of loss to follow-up among clinics which did not trace patients[19], this analysis compared one type of clinic to another rather than comparing outcomes before and after tracing within a single group of patients. Furthermore, while the study from Malawi confirmed a high rate of death among patients who were lost[11], the effect of these deaths on estimates of overall outcomes was not evaluated.

Another difference of this study from the Malawi study[11] is that we analyzed the effects of losses to follow-up on reported risk factors for death after HAART initiation. The tendency of losses to follow-up to bias analyses of risk factors for death, as was documented here for both baseline CD4 counts and male sex, is concerning. While this is less likely to be a problem when analyzing factors with strong biologic associations with survival (like baseline CD4 counts), factors with smaller strengths of association but with considerable public health impact may conceivably be missed if losses to follow-up are censored. Male sex is one example of such a factor. Whereas men have been shown to be at increased risk of death or loss to follow-up after HAART in this study and in several other large cohorts[12], [14], [18], [20], numerous other studies have not found this association[21], [22], including a large multinational analysis from ART-LINC[19]. This finding should be investigated prospectively. More broadly, a critical component of improving outcomes in global antiretroviral therapy scale-up efforts is accurate identification of groups at high risk of death after HAART initiation, and these data indicate the importance of patient tracing in such investigations.

This study has several important limitations. First, reasons for losses to follow-up after HAART initiation were not formally explored, and thus it is impossible to determine from these data the precise interventions capable of keeping patients alive and in care. In particular, we had limited data on social or behavioral factors that may influence adherence[23] to HAART in this setting. Verbal autopsies, which were not performed, may have enhanced the analysis by providing insights into likely causes of death, as has been shown in other sites[22]. Furthermore, while a recent report from Zambia suggested that retention rates among patients lost to follow-up were low despite multiple calls and visits[12], the effectiveness of such efforts needs further prospective evaluation. Another limitation relates to the fact that approximately 5% of patients remained lost to follow-up despite patient tracing. The association between lower baseline CD4 count and being lost to follow-up among patients remaining lost after tracing, which concurs with findings from a large French database[24] and from ART-LINC cohorts not performing tracing[19], suggests that some of these patients also died. If true, the finding of underestimation of deaths and bias due to informative censoring would likely have been more pronounced than was presented here. In addition, although the data from Malawi confirm our estimates of deaths among those lost to follow-up[11], the study was conducted among only approximately 400 adults initiating HAART in 2003 and 2004. As such, results of this study do not necessarily represent the outcomes that are being experienced among patients currently enrolled in the IDCC or in other clinics in Botswana.

Providing data for comparing outcomes after HAART initiation across study settings is one way epidemiologic studies can potentially improve HIV patient care. Using such comparisons, sites with higher mortality or loss to follow-up rates can explore operational characteristics of sites or biologic or behavioral aspects of patients with lower rates in order to attempt to identify procedures or interventions capable of improving patient retention and survival. To facilitate such investigations, survival rates and risk factors for death with minimal inherent bias are needed. Uniform reporting standards have been advocated for randomized clinical trials[25], for analyses of diagnostic tests[26], and, more recently, for observational cohort studies[27], [28]. Adoption of such standards with respect to reporting outcomes from observational cohort studies of HAART use ideally will improve reporting of the number and percent of patients lost to follow-up after HAART initiation, thereby facilitating future research efforts into ways to improve patient care.
